# “I Don't Support It for My Children”: Perceptions of Parents and Guardians regarding the Use of Modern Contraceptives by Adolescents in Arua City, Uganda

**DOI:** 10.1155/2023/6289886

**Published:** 2023-04-03

**Authors:** Godfrey Jalinga Vuamaiku, Joshua Epuitai, Monicah Andru, Mary Aleni

**Affiliations:** ^1^Department of Nursing and Midwifery, Faculty of Health Sciences, Muni University, Arua, Uganda; ^2^Department of Nursing, Faculty of Health Sciences, Busitema University, Mbale, Uganda

## Abstract

Despite the high rates of adolescent pregnancies, the utilization of modern contraceptives is still low among adolescents in Uganda which highlights a missed opportunity for the prevention of unwanted pregnancies among adolescents. We explored the perception of parents and guardians regarding the use of modern contraceptives by adolescents and the roles parents and guardians play in the use of modern contraceptives by the adolescents. A descriptive qualitative study was conducted in one of the suburbs of Arua city in the West Nile subregion in Uganda. Fifteen (15) in-depth interviews were conducted with parents and or guardians to explore their perceptions and roles regarding the use of modern contraceptives by adolescents. Thematic analysis was used in qualitative data analysis. Parents did not support adolescents' use of modern contraceptives. Lack of parental support was related to perceptions that modern contraceptives promote sexual promiscuity, fear that it causes infertility and that it is incompatible with cultural, religious, and moral norms. Parents and guardians opted to emphasize the importance of abstinence, conformity with cultural and religious norms, and the need to focus on completing school instead of encouraging the use of modern contraceptives. Few parents and guardians supported the use of modern contraceptives, specifically condoms, to prevent unwanted pregnancy by the adolescents and parents/guardians, sexually transmitted infections, and early school dropouts. Parents and guardians expressed feelings of inadequacy related to discussions on contraception use with their adolescent children and therefore avoided talking about it. Our study reveals a lack of parental support regarding the use of modern contraceptives among adolescents. Public health interventions which promote intergenerational, socioculturally, and religiously appropriate communication should be instituted in the communities in order to promote sustainable adoption of modern contraceptive use among adolescents.

## 1. Introduction

Adolescent pregnancy is a major public health problem [1]. The World Health Organization (WHO) estimates that about 21 million girls between the ages of 15-19 years get pregnant every year in low and middle-income countries, of which 50% are unintended pregnancies. Approximately 12 million birth occurred in the ages 15-19 years. Data on younger adolescents (below 15 years) was not readily available, but limited data from Angola, Mozambique, Bangladesh, and Nigeria estimates the occurrence of births in this age group at more than 10 per 1,000 girls. [1]. In Uganda, 25% of adolescent girls aged 15-19 were either pregnant or had given birth [2].

Adolescence, which is the period between the age of 10 to 19 years, is characterized by physical, social, psychological, and reproductive changes [1]. The resulting changes place adolescents at a high risk of pregnancy with its associated impacts [2–4]. Adolescent pregnancies have negative health and socioeconomic effects on the mother, child, and the society [2]. Complications of pregnancy and childbirth are the leading causes of maternal mortality and morbidity among adolescent girls from pregnancy-induced hypertensive disorders, labor dystocia, unsafe abortions, and sepsis [1, 2]. Adolescent mothers are immature to take care of a child which in addition to increased risk of preterm birth, low birth weight, and neonatal sepsis among adolescent girls underscores the reduced survival rates among neonates born to adolescent mothers [1]. Adolescent pregnancy is linked to poor social support, stigma, intimate partner violence, inadequate medical care, and financial insecurity [1, 3]. As a result, adolescent mothers may suffer from mental health problems [1, 3]. Adolescent pregnancy is the leading cause of early school dropout which in the long term leads to reduced employment opportunities, deprivation, and poor health and wellbeing of the mother and child [1, 5]. Despite the negative outcomes of adolescent pregnancies, in Uganda, the unmet need for contraceptives among adolescents aged 15-19 stands at 30% and the use of modern contraceptives among adolescents stands at 30% [2] and even much lower among female adolescents at 9.4% [6]. Modern contraceptives, a method or procedure used to prevent pregnancy [7], could be a panacea for the high rates of adolescent pregnancies.

Although studies have identified several barriers against the use of contraceptives among adolescents [6, 8–11], the low use of modern contraceptives among adolescents can also be attributable to a lack of parental guidance, parents' disapproval and fear of retribution related to use of modern contraceptives [12]. Parental disapproval of the use of modern contraceptives among adolescents occurs against the backdrop of cultural and religious fatalistic attitudes and the misconception that modern contraceptives promote promiscuity and the unrealistic expectation that their children are not sexually active [13, 14]. Adolescents are mainly dependent on their parents and guardians for material and psychosocial support, which in turn influences the choices they make [4]. Consequently, the perception of their parents and guardians is significant in their decision to use modern contraceptives and access family planning services [4]. This was evident in Ethiopia where there was increased uptake of modern contraceptives among adolescents whose parents discussed their use with them [15]. Some health workers as well do not support the use of modern contraceptives by adolescents [16] which makes the whole situation more complicated because the health workers are supposed to provide these services to the adolescents. Fear of being judged by the healthcare workers or being seen in the clinic by the community members and consequently being reported to their parent further poses a healthcare system barrier to the use of modern contraception by adolescents [10].

Although parents can play a significant role in the use of modern contraceptives by adolescents, the fact that sex education is considered a taboo let alone nudging adolescents to use modern contraceptives represents a missed opportunity [14, 17]. In 2016, attempts by the Ugandan government to reduce adolescent pregnancies through the introduction of sexuality education in preprimary, primary, and secondary schools by introducing the National Sexuality Framework into the curriculum were opposed by different stakeholders for the fear that the framework was contrary to their believes and values and that it would ruin their children [17, 18].

Currently, most studies related to the use of modern contraceptives have focused on adolescents and health workers [5, 6]. There is limited scholarship on the perception of parents and guardians on the use of modern contraceptives by adolescents in Uganda. Understanding the perception of parents and guardians on the use of modern contraceptives by adolescents will help in developing tailor-made interventions to promote the uptake of modern contraceptives among adolescents. This will in turn reduce the increasing rates of adolescent pregnancies and their associated health and socioeconomic impacts. The aim of conducting this study was to explore the perceptions of parents and or guardians regarding the use of modern contraceptives among adolescents and the roles parents and guardians play in the use of modern contraception by adolescents.

## 2. Materials and Methods

### 2.1. Study Design

We used a descriptive phenomenology study design. This study design allowed the selection of only information-rich respondents who were knowledgeable about or experienced with the phenomenon of interest [17] to express their feelings, perceptions, and beliefs about the use of modern contraceptives by adolescents. This enabled us to get a deeper insight into this area of study.

### 2.2. Study Setting

The study was conducted in Onzivu ward, Arua city which is located in the West Nile subregion in the Northern part of Uganda. Arua city is approximately 500 km from Kampala, the capital city of Uganda. Arua city represents the confluence of ethnic and cultural backgrounds from people in Uganda, South Sudan, and the Democratic Republic of Congo. Onzivu ward is approximately 2.5 km from Arua city center. It is one of the densely populated wards in Arua city, comprising 13 cells with a total number of 12,246 people and about 3,000 adolescents. The socioeconomic status of the families who live in Onzivu varies widely with those in the upper class, middle class, and lower class living together. In the West Nile subregion, 22.4% of adolescents aged 15-19 years have started childbearing [5] representing one of the highest prevalence of adolescent pregnancies in the country.

### 2.3. Study Population

We recruited parents or guardians who had adolescent(s) under their care at the time of data collection and those who have had the experience of caring for an adolescent. Parents and guardians were excluded if informed consent was declined and in cases of a language barrier.

### 2.4. Sampling Procedure

Cluster sampling method was used to select the Onzivu study site from a list of 9 wards in Oluko Division. Data was collected from all the 13 cells of Onzivu ward. The households were identified by the local council, which had one chairman for each of the 13 cells who also acted as a guide and led the data collection team to the respective homes. On average, one respondent was selected from each cell. Purposive sampling was used to recruit participants. This allowed the selection of only information-rich participants to take part in the study as it involved a selection of participants with experience in the upbringing of adolescent children. The sample size was based on the principle of data saturation which in this study was reached after 15 participants were interviewed.

### 2.5. Data Collection Tools and Procedures

In-Depth Interviews (IDIs) were used to collect data. An interview guide with open-ended questions was used to explore the perception of parents and guardians about the use of modern contraceptives by adolescents. The questions in the interview guide focused on gaining insight into the perception of parents and guardians on the use of modern contraceptives by the adolescents and the perceived roles that parents and guardians play in the use of modern contraceptives by adolescents. Sociodemographic information was collected as well (age, sex, tribe, religion, and education level). Each interview with a respondent took a duration of between 20-25 minutes which allowed enough time for each respondent to give his or her views. The interviews were recorded with an audio tape recorder and we also kept a written record of each interview to ensure that all the responses from each participant were taken note. The respondents were interviewed at their respective homes. The respondents were informed before the day of the interview and given the privilege to choose the convenient time for conducting the interview. The data collector went with the home guides who directed them to the home of each of the respondents. The interviews were conducted by the primary author (VGJ), a male, and he was in his final for the award of Bachelor of Nursing Science.

### 2.6. Data Management and Analysis

The audiotaped recording was transcribed verbatim to ensure originality in data [19] and translated into English. Braun and Clarke's thematic analysis was used to analyze the data [20]. The transcribed data was read several times and the underlying concepts were identified. From these concepts, codes, and categories were developed. Broad themes were identified from the codes and categories from the data.

## 3. Results

After each quotation, the identifiers in the brackets represent the following:

IDI respondent: in-depth interview respondent; *n*: the number of respondents with similar responses.

### 3.1. Sociodemographic Characteristics of Respondents

The majority of the respondents were catholic (*n* = 9), aged above 30 years (*n* = 11), female (*n* = 8), and of Lugbara ethnicity (*n* = 9), (Table 1).

Qualitative data analysis identified four themes: lack of parental support, passive parental support, parental approval to use modern contraceptives, and parental roles in sex education and contraceptive use.

### 3.2. Themes

#### 3.2.1. Theme 1: Lack of Parental Support

*(1) Category 1.1: Perceived as a License for Sexual Immorality*. Parents and guardians were opposed to the idea of adolescents using modern contraceptives as the practice was perceived as official permission from parents to allow adolescents to start having sex. Most of the parents and guardians believed that the fear of pregnancy and contracting sexually transmitted infections including HIV were the primary reasons which discouraged adolescents from engaging in sexual practices. As a result, parents believed that the use of modern contraceptives by adolescents would remove the fear factor among adolescents to engage in sex. Most importantly, it was seen to even become worse if an encouragement to use modern contraceptives came from parents or guardians as adolescents would take it as an approval to allow them to have sex. Consequently, respondents believed that the use of modern contraceptives would lead to a sex scandal, addiction, and gross sexual immorality in the communities.

“Telling an adolescent, for example, to begin swallowing things like pills is an indirect way of telling them to go ahead and have sex.” (IDI respondent 8, 38-year-old, female, Catholic).

“It encourages immorality in adolescents” (*n* = 4).

“It teaches these adolescents immorality; how can I tell my children to go out and sleep with men because they cannot get pregnant when they use protection? As a parent, I can't allow.” (IDI respondent 4, 32-year-old, male, Islam).

“Allowing them to use modern contraceptives acts as permission for them to go and have sex the way they want” (IDI respondent 5, 37-year-old female, Catholic).

“They may become addicted to sex because they would know that if they have sex, they will not conceive.” (IDI respondent 6, 29-year-old male, Anglican).

The respondents noted that the possibility of adolescents engaging in unrestricted sexual activity would mean that they would not respect their parents. Because sex is seen culturally to occur in the confines of marriage, adolescents will also see themselves as equals to their own parents/guardians since they would also be doing what their parents/guardians do.

“When adolescents start having sex, they do not even respect their own parents/guardians since they perceive themselves to be mature just like their parents/guardians.” (IDI respondent 9, 46-year-old female, Catholic).

Some parents or guardians did not support the use of modern contraceptives among adolescents because it was attributed to the high incidence of HIV among adolescents. Modern contraceptives were perceived to increase the incidence of HIV by removing the fear of getting pregnant, and indirectly promoting multiple sex partners since most of them do not protect against HIV infection.

“Use of contraceptives like depo provera which does not protect against HIV can also lead to spread of this disease among adolescents simply because they will only focus on protecting themselves against unwanted pregnancies yet HIV also is contracted in this same process.” (IDI respondent 9, 46-year-old female, Catholic).

*(2) Category 1.2: Incompatibility with Cultural, Religious, and Societal Norms*. The use of modern contraceptives by adolescents was seen as the polar opposite in all aspects of culture and religion. This was perceived to compromise and contradict the efforts parents or guardians made in raising children in a decent way consistent with the cultural and religious beliefs, values, and practices. Cultural and religious beliefs were perceived by many as the gold standard for modeling the character and morals of children.

“Culturally, more especially for us Lugbara; be it Maracha, Aringa, Terego, sleeping with men before marriage has impacts on those close relatives even if you do it in secrecy. The categories of people who get these effects are the girl's brothers and the fathers. Like for us the Terego, if a girl is messing outside with men, the father can get frequent fatal accidents or in most cases, the brothers get joint dislocations even in minor activities like playing football. In some homes, it is believed that it even takes away blessings God will give daily like money.” (IDI respondent 1, 40-year-old, male, Catholic).

“Culturally, you model your child to expect the best out of them in terms of behaviors because if the child misbehaves, the blame always comes back to the parents; most especially the mother in case of the girls or females.” (IDI respondent 3, 40-year-old, female, Pentecostal.

“Religiously, it isn't even advisable to encourage children to use modern contraceptives so that they can have sex for as long as they want since the bible doesn't even support sex outside marriage.” (IDI respondent 1, 40-year-old, male, Catholic).

“You as a parent would also like to model your child in the ways of God and use of modern contraceptives is not the ways in which you can instill a Christian virtue in your children” (IDI respondent 3, 40-year-old, female, Pentecostal).

Some of the respondents thought that it was not morally right to tell their children to use modern contraceptives:

“No parent in their right mind will say that adolescents should use modern contraceptives”. (IDI respondent 11, 35-year-old female, Catholic).

“It is not good. I don't support it for my children. Others can go for them but not my own children”. (IDI respondent 12, 38-year-old male, Anglican).

*(3) Category 1.3: Fear of Side Effects*. Fear of side effects was one of the major reasons brought out by respondents for not supporting the use of modern contraceptives by adolescents. Respondents expressed reservations about the use of modern contraceptives by adolescents because it was thought that modern contraceptives would cause infertility and congenital anomalies in the long term.

“These things badly affect them in the future and so many people have realized this” (n =2, IDI respondent 2, 52-year-old male, Anglican, IDI respondent 11, 35-year-old female, Catholic).

At times I will not support the use of modern contraceptives because they have side effects on girls. For example, if it stays for long, the child may give birth to a baby who is abnormal” (*n* = 6).

“As for me as a parent, I don't support it at all. It is not good because these things have serious side effects”. (*n* = 3).

“So, if we who have given birth to children fear the side effects, how can I allow my child to go for such things when I know that it will affect her in the future?”(IDI respondent 3, 40-year-old female, Pentecostal).

“I don't like the idea, because even we the adults, sometimes we face challenges with these things (experiences side effects); now what about these who have not produced any children? In case that thing blocks them for good [causes infertility]” (IDI respondent 9, 46-year-old female, Catholic).

Related to perceived side effects, myths and misconceptions surrounding the use of modern contraceptives was one of the major reasons for the parents' disapproval of adolescents' use of modern contraceptives.

“It may kill their cells and may stop them from producing.” (IDI respondent 6, 29-year-old male, Anglican).

“Specifically for ladies concerning the injectable contraceptive, getting these injections at a time when somebody has not had children is not good since these contraceptives may kill their cells and cause them not to produce in future.” (IDI respondent 9, 46-year-old female, Catholic).

“…so what we believe is, people say that when they take these modern contraceptives, they have worse side effects in future more especially affecting their uterus and a lot of complications but as I said, clinically, I do not really know much about the details of these side effects. If I am to know the truth of the matter, it could guide me in guiding them very well to make right decisions.” (IDI respondent 8, 38-year-old female, Catholic).

*(4) Category 1.4. No Need for Modern Contraceptive*. The majority of the respondents who were opposed to use of modern contraceptives by adolescents did not see any justifiable reason for it.

“I don't think if it's right for them to use protection [condoms]” (*n* = 2).

“There's no need since girls are not supposed to sleep with men.” (*n* = 3).

“Not at all, I don't see any reason as to why the adolescents should use modern contraceptives” (IDI respondent 6, 29-year-old male, Anglican).

#### 3.2.2. Theme 2: Passive Parental Support

Although parents were reluctant to allow their adolescents to use modern contraceptives, a few admitted that modern contraceptives maybe necessary given the breakdown of societal values and morals. Although parents did not talk to their children regarding using modern contraceptives, the respondents were passively resigned to allowing adolescents to use modern contraceptives. Passive acceptance of modern contraceptives was related to perceived “unruly” behaviors of adolescents, recognition that adolescents were sexually active, and the need to prevent unwanted pregnancy.

“For us, we don't talk about it but it is good because it protects girls from unwanted pregnancies.” (IDI respondent 7, 34-year-old female, catholic).

“As a parent, because these girls are stubborn and so, especially during this lockdown. Those who are above 18 can use modern contraceptives just to protect them against unwanted pregnancies so that when school begins, they can resume.” (IDI respondent 3, 40-year-old female, Pentecostal guardian of 2 adolescents).

“Well, the idea is good but I haven't gone so deep to understand the impacts of contraceptives because in rumors, we have heard that these contraceptives have effects on the ladies but clinically, I haven't gone so deep to know the details.” (IDI respondent 4, 32-year-old male, Islam).

“Adolescents are so active groups of people, ruling them may not be easy because they go as they want. According to my understanding, injecting people with these drugs as said by other people also have side effects. This is my worry. If this wouldn't have been the case, I would just say they (adolescents) go ahead and get them.” (IDI respondent 6, 29-year-old male, Anglican).

#### 3.2.3. Theme 3: Parental Approval to Use Modern Contraceptives

Some parents or guardians admitted that adolescents can use certain methods of modern contraceptives. Parents and or guardians perceived condoms, as the only method of modern contraceptives which could be recommended for use by adolescents. Condoms were approved for use among adolescents because of the perceived lack of side effects.

“I can now accept it [condoms] because it leaves them free; the girl just takes her way and also the boy in a healthy way with no effects in the future. So, as for that, I accept only that method [condoms]. If only the other methods are also as safe as condoms without future side effects, then I would have no problem accepting them. But as for condoms, I even accepted it as soon as it was introduced in the country.” (IDI respondent 2, 85-year-old male, Anglican, 17 children).

“If only the other methods are also as safe as condoms without future side effects, then I would have no problem accepting” (IDI respondent 9, 46-year-old female, Catholic, IDI respondent 14, 29-year-old female, Catholic).

Parents who approved the use of modern contraceptives among adolescents cited contraceptive benefits as the reasons why adolescents would use modern contraceptives including prevention of unwanted pregnancy, sexually transmitted infections including HIV, early school dropouts, and cases of rape.

“To protect adolescents from unwanted pregnancies, also prevention of sexually transmitted infections.” (IDI respondent 3, 40-year-old female, Pentecostal).

“They can get pregnant, drop out of school, and even get AIDs” (IDI respondent *n* = 3),

“…it helps adolescents to prevent teenage pregnancy. You know when ladies are very young and you don't guide them via the use of contraceptives, they can easily conceive and it affects their education and so on. So, for the sake of them continuing in pursuit of their studies, they can use modern contraceptives to prevent teenage pregnancies.” (IDI respondent 8, 38-year-old female, Catholic).

Another respondent who had not supported the use of modern contraceptives by adolescents in normal situations believed that its use by adolescents could be justified in special scenarios such as rape to prevent unwanted pregnancy.

“Special scenarios like cases of rape where contraceptives, just as postexposure prophylaxis is administered to prevent pregnancies. But other than that, I can't allow my girls to get any method of modern contraceptives.” (IDI respondent 5, 37-year-old female, Catholic).

#### 3.2.4. Theme 4: Parental Role in Sex Education and Contraceptive Use

*(1) Category 4.1: Instilling Cultural and Religious Values*. Instead of encouraging adolescents to use modern contraceptives, some parents or guardians believed that instilling and modeling good cultural and or religious norms was a superior option. Teaching children right from childhood virtues of abstinence, the importance of school, and resisting sexual advances till marriage was seen to nullify the need to use modern contraceptives by adolescents.

“It depends on the kind of children you have. As a parent, if you model your children well from the beginning, even if they reach the age of adolescence, they will abstain.” (IDI respondent 6, 29-year-old male, Anglican).

“I tell my children to focus on their studies and avoid things that distract them. I also teach them good morals from the start and expect them to be humble children. I also train my children in the ways of God to make them become morally upright people in life.” (IDI respondent 7, 34-year-old female, Catholic).

“When those ladies were still under my care, when they were in school, I did not advise them to use any modern contraceptives. What I used to tell them is “be loyal and faithful, follow your religion, and follow whatever I tell you.” (IDI respondent 8, 38-year-old female, Catholic).

“Teaching them the cultural ways of life and the effects of sexual intercourse during adolescence such as how sex during this period may result in pregnancy and also sexually transmitted diseases. This will make them fear and abstain.” (IDI respondent 1, 40-year-old male, Catholic).

*(2) Category 4.2: Supporting the Use of Contraceptives*. Sex was considered a sensitive topic, and as a result, parents did not directly encourage their adolescent children to use modern contraceptives. Instead, parents did not restrict adolescents who were interested to use modern contraceptives.

“Leaving them free (not prohibiting use) by parents to use the modern contraceptives” (*n* = 2, IDI respondent 3, 40-year-old female, Pentecostal, IDI respondent 12, 34-year-old male, Anglican).

“Limiting them or allowing them to go ahead with it, though allowing them as a parent would imply that you're only concerned about pregnancy but not the morals.”(IDI respondent 4, 32-year-old male, Islam).

“I so much recommend the use of condoms.” (IDI respondent 2, 85-year-old male, Anglican).

A few respondents admitted that they go as far as creating awareness for their adolescents on modern contraceptives. To them, making adolescents aware of modern contraceptives is not directly telling them to go and use but empowers them with the knowledge to guide them in making informed decisions. These are of parents or guardians who believed that times have changed and the guarantee that culture and religion alone would play a role in limiting the adolescents from having sexual relationships is a total delusion. However, those who admitted to playing such a role were those who were educated and mainly professionals in the medical field. This particular finding could also offer insight into how the knowledge parents/guardians have on modern contraceptives affects the roles play in their use by adolescents.

“Always speaking to them about the different types of modern contraceptives, their advantages and its goodness. It is better for them to be aware the contraceptives so they can make the right choices. These are not the times of our forefathers where cultural values were considered very important and many children no longer value our culture.” (IDI respondent 3, 40-year-old female, Pentecostal).

“Even after giving them knowledge of modern contraceptives, it is still possible to impact in them the cultural virtues and religious values that can make them decide whether they can go for sex or not.” (IDI respondent 9, 46-year-old female, Catholic, IDI respondent 12, 34-year-old male, Anglican).

*(3) Category 4.3: Emphasis on Education*. Parents who were opposed to the idea of adolescents using modern contraceptives thought that emphasizing the dangers of early indulgence in sexual activities will instill fear among their children and echo the consequences of engaging in sex, especially the risk of contracting sexually transmitted infections including HIV, while other parents opted to emphasize the importance of completing school first before engaging in sex. Parents believed that instilling fear would discourage adolescents from engaging in sex.

“As a mother, I can't advise my children to go for those things. They should wait for the right time with their own husbands after getting married and when they are married, no need for protection [to use condoms] since that is their own husband [they're having sex with their husband].” (IDI respondent 15, 37-year-old male, Catholic).

“I tell my children to focus on their studies and avoid things that distract them from getting pregnant.” (IDI respondent 11, 35-year-old female, Catholic).

*(4) Category 4.4: Difficult to Educate on the Use of Modern Contraceptives*. A few parents who educate their adolescents on issues of contraceptives and sexuality admitted that it was the most difficult thing for a parent or guardian. The respondents believed that healthcare workers with expertise and knowledge of contraception would be best suited to talk to their adolescents regarding sexuality and contraception use. Parents acknowledged that the efforts they have made were giving basic education on sexuality education but do not have enough knowledge in the area of contraceptive use.

“As parents, the most difficult thing for us to do is talking to the adolescents about the use of modern contraceptives. We do not know what to do about it. If knowledgeable people can reach out to them and speak to them about this subject, otherwise, the little I have always told my own is to ensure they use condoms correctly in case they have got their own girlfriends and also consider testing for HIV.” (IDI respondent 9, 46-year-old female, Catholic).

## 4. Discussion

The study explored the perceptions of parents and guardians and the roles they play regarding the use of modern contraceptives among adolescents. The study findings reveal that parents or guardians were not in support of adolescents using modern contraceptives. Parental disapproval was related to the perceived implied permission that contraception would promote sexual promiscuity, incompatibility with religious and cultural beliefs, and fear of the side effects, and was seen as a violation of moral imperatives. However, some parents approved the use of contraceptives in cases of rape, prevention of unwanted pregnancy, and sexually transmitted infections. Condoms were the most preferred contraception because of the perceived lack of side effects as compared to other methods of contraception. Despite the strong opposition to allowing adolescents to use contraception, parents were resigned to allow their adolescents to use contraception related of the practical difficulty of modeling children. While parental education on sex and contraception use was considered taboo in many African countries [21, 22]. Parents opted not to emphasize the use of modern contraception but instead preferred to talk about the need to abstain and advise adolescents on the punitive consequences of engaging in sexual activities. These findings are related to the Ugandan context where the fertility rates are high due to early initiation of childbearing at a young age, high teenage pregnancy rates, and low utilization of contraceptives among adolescents [2, 8, 23].

Although 62% of women have their sexual debut by the age of 18 in Uganda [2], parents or guardians did not acknowledge the fact that adolescents were sexually active and consequently need to use modern contraception. Self-serving bias may account for the fact that parents were aware that adolescents in general were sexually active and yet were unwilling to admit it among their own adolescent children. Previous studies have reported the misconception that modern contraceptives promote sexual promiscuity [4, 6]. As a result, parents thought that encouraging unmarried young adolescents to use modern contraceptives was implicitly permitting adolescents to start engaging in sexual practices.

The use of modern contraceptives was perceived to remove the safety net and fear of getting pregnant which was consistent with a study that noted that prevention of unwanted pregnancy was the primary motivation for using contraception among adolescents [6, 7]. Consequently, the removal of the fear factor was seen to promote unbridled sexual activity and increased the risk of acquiring HIV. These perceptions were shaped by religious, cultural, and societal moral beliefs which attached high value to sex in the context of marriage, but also prevalent notions that the use of modern contraceptives was not consistent with many cultures in Uganda evidenced by the modern contraceptive prevalence rate of 35% among married women and 47% among unmarried women [2].

Studies have underscored the fear of side effects as the major deterrent to the use and early discontinuation of modern contraceptives [8, 9]. In our study, the most feared side effect was the perceived likelihood that contraceptives could cause infertility, a finding which was consistent with previous studies [9]. As such, parents were strongly opposed to allowing adolescents to use contraceptives for fear of infertility for their adolescent children. The misconception is a powerful deterrent given that in Uganda, like most African cultures, children are highly valued as a source of wealth, domestic labor, and social security at old age [10, 24]. For women whose status in society is tied to their ability to bear children, modern contraceptives were perceived to deny women the privilege to produce children. Counselling parents would be instrumental in addressing these misconceptions and myths about contraception use and, consequently, promoting the utilization of modern contraceptives among adolescents.

Some of the respondents were passively resigned to allowing adolescents to use modern contraception out of the recognition that adolescents were sexually active, unruly, and unable to conform to societal norms and expectations of sexual chastity. Parents or guardians supported the use of modern contraceptives passively by not speaking against its use as well as not stopping adolescents who chose to use a particular method of contraception. This underscores parents' reservations, reluctance, and laissez-faire attitudes in regard to adolescents' use of modern contraceptives. Male condoms were the only approved method of contraception among adolescents which may relatively be less effective given the inconsistency with the use in the long-term and reliance on the male partner. In Kenya, parents were resigned to allowing adolescent girls who had started childbearing to use contraception [9], which indicates inherent opportunities to advance the use of modern contraceptives even in the face of parental opposition. Parental approval of adolescents to use modern contraceptives occurred in the backdrop of the motivation to prevent unwanted pregnancies, sexually transmitted infections, and need to enable adolescent girls to complete school. Interventions that raise awareness of the fact that adolescents are sexually active, and emphasize the inherent contraceptive benefits of preventing pregnancy may be successful in promoting the adoption of modern contraceptives.

Sex and contraception education is considered taboo in most African cultures [14, 17, 25–28] which is consistent with the reluctance shown by parents or guardians in our study to advise their adolescent children to use contraceptives. In addition, the respondents noted a lack of confidence, inadequacy, and low self-efficacy to provide education regarding contraception, which leaves adolescents vulnerable to unreliable influence from the media and their peers [13, 14]. Failure to provide sex education occurs in the backdrop of parents' failure to recognize the negative implications of failing to provide sex and contraception education to adolescents [27]. In Kenya, parents provided sex and contraception education to their adolescents when teenage pregnancy was reported in the neighborhood or following rumors that their adolescent children were in a romantic relationship [11]. Sex education was often focused on instilling fear, and was limited to abstinence, and the need to focus on studies. Conformity with religious and cultural norms was perceived to nullify the need for modern contraceptives. Sex education in schools including encouraging adolescents to use modern contraceptives was opposed in Uganda as it was perceived as promoting sexual practices including same-sex relationships among adolescents [17]. Uganda has primarily relied on abstinence-only messages in the prevention of adolescent pregnancy and HIV transmission among the unmarried population [17]. However, studies have shown that abstinence-only messages and fear-leaning messages are ineffective in promoting sexual chastity let alone preventing adolescent pregnancies. [10, 13]. Therefore interventions targeting important stakeholders in the lives of adolescents such as parents, guardians, and community leaders need to be geared towards dispelling myths and misconceptions regarding contraceptive safety through educational campaigns and community engagements [29].

## 5. Limitation of the Study

Though parents/guardians of different ethnicities and gender were interviewed, the respondents' narratives might have been biased by the interviewer being young, a student, and not having parenting experience of adolescents. This could have prevented discussions of some of the important views and parental roles regarding the use of modern contraceptives in adolescents.

## 6. Conclusions

Our findings show that parents and guardians did not approve of the use of contraceptives among adolescents. Disapproval of contraceptive use was related to fear of side effects especially infertility, the perception that using modern contraceptives promotes sexual promiscuity, and that the use of contraceptives among adolescents was incompatible with religious, cultural, and moral norms and beliefs. Parents in a few instances allowed the use of contraceptives for the prevention of unwanted pregnancies, sexually transmitted infections, early school dropouts, and cases of rape. Parental and guardian approval of modern contraceptive use among adolescents was in form of not prohibiting its use, and not speaking against the use of contraceptives among adolescents. Parents or guardians expressed difficulty and inadequacy to openly discuss contraception use with their adolescents, while the limited education on contraception use focused on abstinence, instilling fear of contracting sexually transmitted infections, and the need to complete studies. Parents and guardians play a central role in the adoption of modern contraceptive use among adolescents. Therefore interventions that promote intergenerational, socioculturally, and religiously appropriate communication need to be instituted in the communities in order to promote sustainable adoption of modern contraceptive use among adolescents.

## Figures and Tables

**Table 1:** Sociodemographic characteristics of respondents.

Variable	Frequency (*n* = 15)	Percentage
*Age*		
20-30	4	26.70
31-40	9	60.00
>40	2	13.30
*Sex*		
Female	8	53
Male	7	47
*Religion*		
Catholic	9	60.00
Anglican	4	27.66
Islam	1	6.66
Pentecostal	1	6.66
*Tribe*		
Lugbara	9	60.00
Langi	1	6.67
Alur	4	26.66
Kakwa	1	6.67
*Level of education*		
Primary	1	6.67
Secondary	5	33.33
Tertiary	9	60.00

## Data Availability

The primary data used to support the findings of this study are available from the corresponding author upon request.
